# Clinical characteristics of 104 patients with cluster headache: a single-center cross-sectional study

**DOI:** 10.3389/fneur.2026.1993055

**Published:** 2026-10-30

**Authors:** Furong Li, Xin Pan, Xiaowen Sui, Junmin Tao, Shuhan Liu, Yanan Zhang, Weiwei Dong, Hongling Zhao

**Affiliations:** 1 Stroke Center, Central Hospital of Dalian University of Technology (Dalian Municipal Central Hospital), Dalian, China; 2 Neurology Department, Central Hospital of Dalian University of Technology (Dalian Municipal Central Hospital), Dalian, China

**Keywords:** accompanying symptoms, clinical features, cluster headache, gender ratio, psychological comorbidity

## Abstract

**Objective:**

To analyze the clinical characteristics of patients with cluster headache (CH).

**Methods:**

Between January 2021 and March 2026, a total of 135 patients with suspected CH who first visited the headache outpatient clinic at Central Hospital of Dalian University of Technology (Dalian Municipal Central Hospital) for headache attacks were enrolled. After excluding 31 patients who did not meet the eligibility criteria (secondary headache or incomplete data), the clinical data of the remaining 104 patients were analyzed.

**Results:**

(1) All 104 patients completed the follow-up, with a male-to-female ratio of 4.2:1, and 97.11% were diagnosed with episodic CH (eCH). (2) Depression and anxiety were significantly more prevalent in females (*p* = 0.004 for both), and Sleep disorders also showed a significantly higher prevalence in females (70.00% vs. 42.86%, *p* = 0.029). (3) Among male patients, right-sided attacks were the most common laterality (54/84, 64.29%), whereas among female patients, left-sided attacks predominated (14/20, 70%). Alternating bilateral attacks occurred exclusively in four female patients (20% of females). (4) A circadian pattern was observed in 82.69% of patients, and the predominant time of onset was the afternoon (12:00–17:00). (5) 7.69% (8/104) of patients experienced no autonomic symptoms; their accompanying features included agitation with nausea, agitation with phonophobia/photophobia, or agitation alone.

**Conclusion:**

This study provides a comprehensive analysis of the clinical characteristics, comorbidity patterns, attack features, and accompanying symptoms of CH in the Dalian region. It enhanced local physicians’ awareness of cluster headache, which may help reduce misdiagnosis, shorten the time to diagnosis, and improve patients’ quality of life.

## 1 Introduction

Cluster headache (CH) is one of the most severe and disabling pain disorders in humans. It is the most common type of autonomic cephalalgia and a relatively rare primary headache. CH manifests as recurrent, severe, unilateral headaches accompanied by ipsilateral autonomic symptoms, restlessness, or agitation, or both, causing significant distress and disruption to daily life (1, 2). The prevalence of CH in the general population is approximately 0.1%, with an incidence rate ranging from 2.07 to 9.8 per 100,000 person-years. The onset typically occurs before the age of 50 years, with a peak incidence between 20 and 29 years (3). CH can be classified as episodic CH (eCH), chronic CH (cCH), and probable CH (2).

Epidemiological studies have shown that approximately 21% of patients experience unemployment due to CH, and 94% of those with cCH report restricted occupational activities during attacks. Even during remission, 15% of patients continue to experience limitations in their daily activities. The estimated annual total costs were €6,321 for patients with eCH and €20,967 for those with cCH (4). Furthermore, comorbidities such as depression, anxiety disorders, and suicidal tendencies are closely associated with CH (5–7), imposing significant psychological burdens and underscoring the urgent need for effective treatments.

Given the relative rarity of CH and the limited diagnostic capacity among physicians, this study aimed to analyze the clinical characteristics of patients with CH attending our headache specialty clinic. To our knowledge, this is the first single-center cross-sectional study to comprehensively characterize the clinical profile of CH in the Dalian region, providing the first evidence of sex-specific features in headache laterality and psychiatric comorbidities, and a trend toward earlier age at onset in females, as well as identifying patients presenting without cranial autonomic symptoms.

## 2 Methods and materials

### 2.1 Patient selection

A total of 135 patients with suspected CH who first visited the headache outpatient clinic at Dalian University of Technology Affiliated Central Hospital (Dalian Central Hospital) due to headache attacks between January 2021 and March 2026 were enrolled. Of these, 31 patients were excluded because they did not meet the study criteria (secondary headache or incomplete data). The remaining 104 patients were all diagnosed with CH according to the International Classification of Headache, Third Edition (ICHD-3) (Figure 1).

**Figure 1:**
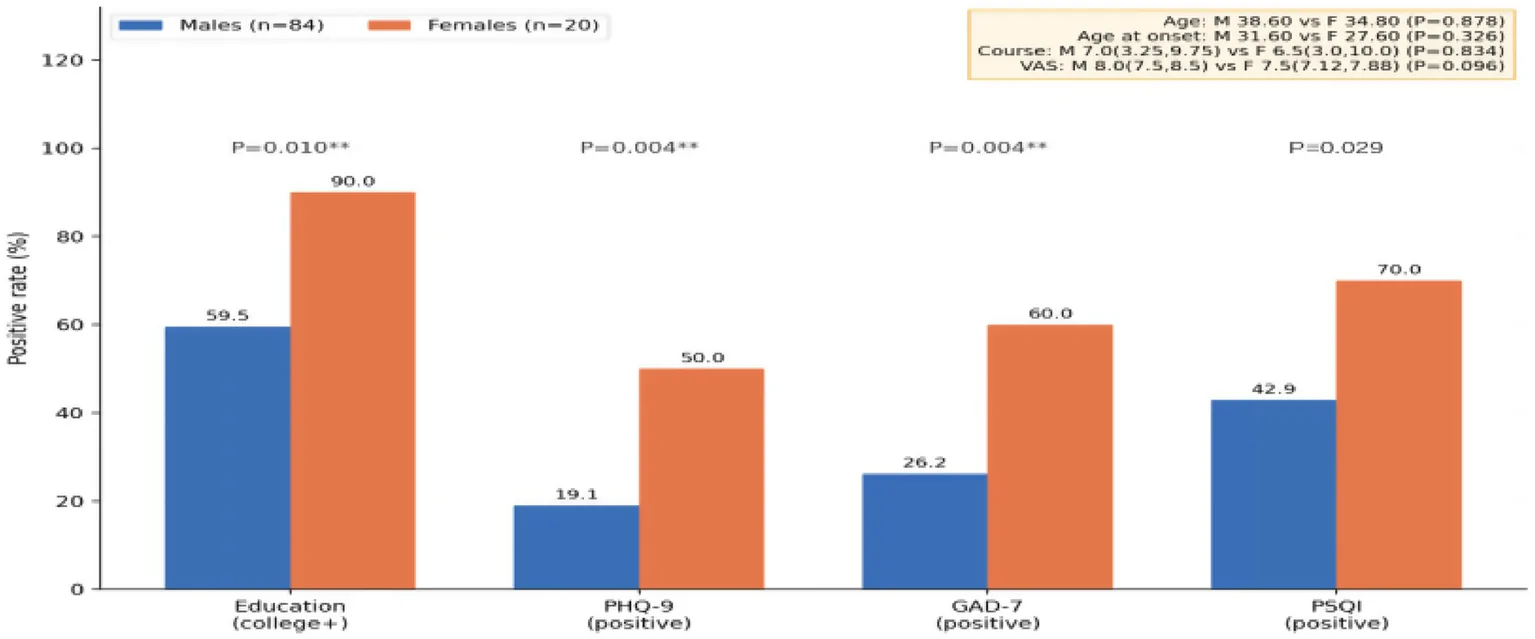
Sex-specific differences. Sex-specific differences in education, depression, anxiety, and sleep disorders. PHQ-9 ≥ 10 indicates depression; GAD-7 ≥ 10 indicates anxiety; PSQI ≥ 8 indicates sleep disorder. *p* values from Chi-square test.

Diagnostic criteria for CH (2): According to the ICHD-3, CH is defined as severe or very severe unilateral pain localized to the unilateral orbit, supraorbital region, and/or temporal regions. During an attack, headache is accompanied by at least one of the following symptoms or signs: (a) conjunctival injection and/or lacrimation, (b) nasal congestion and/or rhinorrhea, (c) eyelid edema, (d) forehead and facial sweating, and (e) miosis and/or ptosis. The pain typically lasts 15–180 min, with an attack frequency ranging from once every other day to up to eight times a day.

eCH is characterized by episodes occurring within a cluster period that lasts from 7 days to 1 year, followed by a headache remission phase lasting at least 3 months.

cCH is defined as the absence of a remission period or a remission period shorter than 3 months that persists for at least 1 year.

The misdiagnosis time was defined as the interval between the first CH attack and a correct diagnosis. Patients diagnosed correctly during the first cluster period were assigned a misdiagnosis time of 0.

Attack duration was defined as the typical duration of an untreated attack, as reported by each patient.

*Exclusion criteria*: consideration or coexistence secondary headache.

### 2.2 Questionnaire

All patients were diagnosed by at least two neurologists, one with specialized training in headache. Face-to-face questionnaires and scale assessments were administered by two professionally trained nurses at the first visit, with one nurse primarily interviewing the patients and the other assisting with questionnaire distribution and collection. The assessment covered the following domains: ① demographics: sex, age, disease duration, education, age at onset, time to diagnosis, lifestyle (smoking, alcohol), family history and comorbid headaches, and psychiatric comorbidities (anxiety, depression, and sleep disorders). ② Attack characteristics: nature, intensity, location, frequency, duration, circadian pattern, nocturnal onset, and accompanying symptoms. ③ Cluster period characteristics: duration and frequency. Pain intensity was measured using the Visual Analog Scale (VAS).

Mental health status was assessed using the validated Chinese version of the Generalized Anxiety Disorder 7-item Scale (GAD-7) and the Patient Health Questionnaire 9-item Scale (PHQ-9), both of which are widely used in patients with headache. Scores greater than 10 on either scale was considered indicative of anxiety or depression, respectively (8).

Sleep disorders were assessed using the Pittsburgh Sleep Quality Index (PSQI). A score of ≥ 8 was adopted as the threshold, as it captures not merely poor sleep quality but clinically meaningful sleep impairment (9).

### 2.3 Statistical analysis

Statistical analyses were conducted using SPSS 23.0. The normality of continuous variables was assessed using the Shapiro–Wilk test. Variables conforming to a normal distribution were expressed as mean ± standard deviation (SD) and compared between two groups using the independent-samples t-test, whereas those not conforming to a normal distribution were presented as median with interquartile range [M (P₂₅, P₇₅)] and analyzed using the Mann–Whitney U test. Categorical variables were summarized as counts (percentages) and compared using the Chi-square test; Fisher’s exact test was applied when the expected frequency in any cell was less than 5. Binary (univariate) analysis was first performed to screen for potential factors associated with cluster headache, followed by multivariable logistic regression analysis to identify independent associated factors, with odds ratios (ORs) and 95% confidence intervals (CIs) calculated. A two-sided *p* < 0.05 was considered statistically significant.

## 3 Results

A total of 135 patients with suspected CH presenting with headache attacks were initially screened, of whom 31 were excluded from the study. Ultimately, 104 patients completed follow-up and were included in the analysis. These 104 patients underwent a cluster headache questionnaire and scale assessment conducted jointly by two nurses, which also included assessment of other comorbid primary headaches.

### 3.1 Demographic and clinical characteristics

The cohort comprised 84 males and 20 females, yielding a male-to-female ratio of 4.2:1. eCH accounted for 97.11% of cases (*n* = 101), while cCH accounted for 2.89% (*n* = 3). A positive family history of CH was reported in 8 patients (7.69%), all of whom were first-degree relatives; nevertheless, no genetic testing was conducted in these cases. No patient had comorbid tension-type headache, migraine, or trigeminal neuralgia.

The mean age at onset was 30.83 ± 9.16 years, and the mean disease duration was 7.0 (3.0,10.0) years (Mann–Whitney U test). The age at onset was higher in males than in females (31.60 ± 8.47 vs. 27.60 ± 11.61), although the difference was not statistically significant (*p* = 0.326). The mean VAS scores for all patients were 8.00 (7.50,8.50), with no significant sex difference [males vs. females: 8.0 (7.5, 8.5) vs. 7.5 (7.12,7.88); *p* = 0.096] (Mann–Whitney U test).

Regarding lifestyle factors, 18 patients (17.31%) were current smokers with a history of smoking, with a significantly higher prevalence in males [18 (21.43%) vs. 0 (0.00%), *p* = 0.021]. Light drinking consumption (<3 times/week, one standard drink per occasion) was reported by 28 patients (26.92%), with a higher proportion in males (26/84, 30.95%) than in females (2/20, 10.00%); however, this difference did not reach statistical significance (*p* = 0.058). The proportion of patients with a college education or higher was greater among females than males [18 (90.00%) vs. 50 (59.52%), *p* = 0.010].

The prevalence of depression (PHQ-9) was significantly higher in females than in males (50.00% vs. 19.05%, *p* = 0.004), as was the prevalence of anxiety (GAD-7) (60.00% vs. 26.19%, *p* = 0.004). Sleep disorders (PSQI ≥8) were more prevalent in females than in males (70.00% vs. 42.86%, *p* = 0.029).

In the multivariable logistic regression analysis with CH diagnosis as the dependent variable and adjustment for age and sex, GAD-7 positivity showed an OR of 4.32 (95% CI: 0.99–18.86; *p* = 0.051), suggesting a trend toward an association between anxiety and CH; however, the association did not reach statistical significance (*p* = 0.051).

These findings are summarized in Table 1 and Figure 1.

**Table 1:** Demographic, clinical, and psychological characteristics of patients with CH.

Binary (univariate) analysis of factors				
Patient characteristics	Total (*n* = 104)	Males (*n* = 84)	Females (*n* = 20)	*p* value
Age at onset, years	30.83 ± 9.16	31.60 ± 8.47	27.60 ± 11.61	0.326
Age, years	37.87 ± 9.82	38.60 ± 8.95	34.80 ± 12.96	0.226
Disease duration, years	7.0 (3.0,10.0)	7.0 (3.25,9.75)	6.5 (3.0,10.0)	0.834
Pain intensity (VAS)	8.00 (7.50,8.50)	8.0 (7.5,8.5)	7.5 (7.12,7.88)	0.096
Smoking status, *n* (%)				0.021
Smoking	18 (17.31)	18 (21.43)	0 (0)	
No	86 (82.69)	66 (78.57)	20 (100.00)	
Drinking, *n* (%)				0.058
No	76 (73.08)	58 (69.05)	18 (90.00)	
Light drinking	28 (26.92)	26 (30.95)	2 (10.00)	
Family history of CH, *n* (%)				0.648
Yes	8 (7.69)	6 (7.14)	2 (10.00)	
No	96 (92.31)	78 (92.86)	18 (90.00)	
Educational level, *n* (%)				0.010
College or higher	68 (65.38)	50 (59.52)	18 (90.00)	
Junior high school or lower	36 (34.62)	34 (40.48)	2 (10.00)	
PHQ-9 scale, *n* (%)				0.004
No	78 (75.00)	68 (80.95)	10 (50.00)	
Yes	26 (25.00)	16 (19.05)	10 (50.00)	
GAD-7 scale, *n* (%)				0.004
No	70 (67.31)	62 (73.81)	8 (40.00)	
Yes	34 (32.69)	22 (26.19)	12 (60.00)	
PSQI, *n* (%)				0.029
No	54 (51.92)	48 (57.14)	6 (30.00)	
Yes	50 (48.08)	36 (42.86)	14 (70.00)	
PHQ-9 ≥ 10 indicates depression; GAD-7 ≥ 10 indicates anxiety; PSQI ≥8 indicates sleep disorder. Light drinking: regular consumption of <3 times/week, one standard drink (12 g pure alcohol) per occasion. VAS, Visual Analog Scale; CH, cluster headache.				

Multivariable logistic regression analysis of factors		
Variable	OR (95% CI)	*p* value
VAS score (per +1 point)	0.50 (0.22–1.13)	0.097
Smoking (yes vs. no)	0.09 (0.00–1.63)	0.102
Family history (yes vs. no)	3.36 (0.55–20.41)	0.189
Education (college or higher vs. Junior high schoolor lower)	4.08 (0.82–20.39)	0.087
PHQ-9 (yes vs. no)	1.03 (0.21–5.09)	0.973
GAD-7 (yes vs. no)	4.32 (0.99–18.86)	0.051

Dependent variable: diagnosis of cluster headache (yes vs no). Adjusted for age and sex. OR, odds ratio; CI, confidence interval; VAS, Visual Analog Scale; PHQ-9, Patient Health Questionnaire-9; GAD-7, Generalized Anxiety Disorder-7.

### 3.2 Misdiagnosis time

After excluding four patients who received a definite diagnosis at their first episode, the median time to correct diagnosis for the remaining 100 patients 7.0 years (IQR: 4.0–10.0 years). Among these 100 patients, 58.9% experienced a diagnostic delay exceeding 5 years and 9.8% received a correct diagnosis within one year (Figure 2).

**Figure 2:**
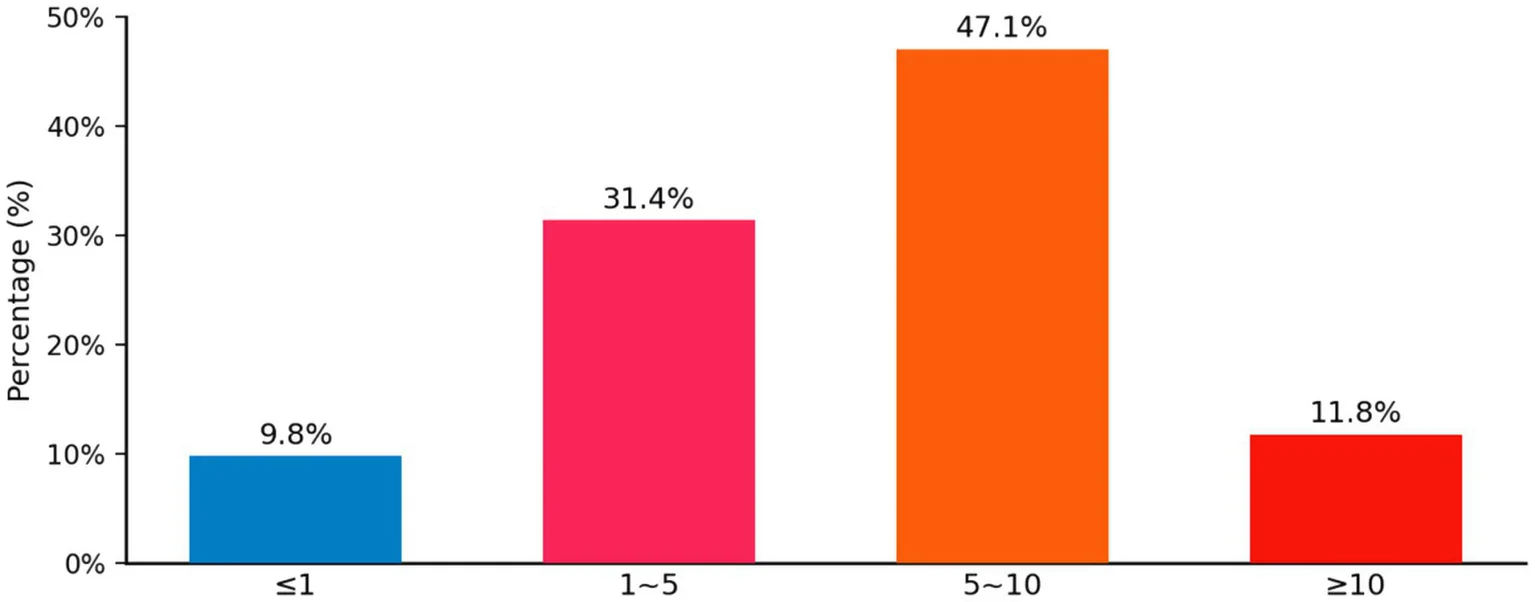
Time delay for diagnosis (years). Time from first attack to correct diagnosis (*n* = 100). Four patients diagnosed at their first episode were excluded.

### 3.3 Characteristics of individual headache attacks

The median attack duration was 60 min (IQR: 30–101 min; overall range, 20 min to 4 h). In the absence of acute treatment, six patients (5.77%) experienced attacks lasting more than 3 h, relief within 2 h was achieved in 84.62% (88/104) of patients, and within 1 h in 61.54% (64/104). Regarding attack frequency, the most common pattern was one episode per day, observed in 46.15% (48/104) of patients.

Right-sided headache was the most common laterality overall (56/104, 53.85%) and predominating in males (54/84, 64.29%), whereas left-sided headaches predominated in females (14/20, 70.00%). Four patients (all females) experienced alternating bilateral headaches, and this sex difference was statistically significant (*p* <0.001).

The mean VAS pain score was 8.00 (7.50, 8.50). The most frequent pain location was the orbital region (96.15%), followed by the forehead (86.54%). Pain involving the cheek and dental region was uncommon, observed in only 2 patients (1.92%), both of whom experienced concurrent pain at both sites. The predominant pain quality was distending pain (96.15%), followed by throbbing pain (40.38%).

The findings are detailed in Tables 2, 3.

**Table 2:** Clinical characteristics of individual headache attack.

Clinical characteristics	Statistic
Median duration of a headache attack (min)	60 (30 ~ 101)
Attack duration (h)	
≤ 1	64 (61.54)
> 1 and ≤ 2	24 (23.08)
> 2 and ≤ 3	10 (9.62)
> 3	6 (5.77)
Attack frequency within cluster period (times/day)	
≥2	44 (42.31)
1–2	6 (5.77)
1	48 (46.15)
Once every other day	6 (5.77)
Attack during sleep	
All	8 (7.69)
1–3 times/10times	16 (15.38)
4–6 times/10times	12 (11.54)
7–9 times/10times	12 (11.54)
No	56 (53.85)
Pain intensity (VAS)	8.00 (7.50, 8.50)
Laterality of the headache in this cluster bout	
Left-side only	44 (42.31)
Right-side only	56 (53.85)
Alternating bilateral	4 (3.85)
Nature of pain	
Distending pain	100 (96.15)
Throbbing pain	42 (40.38)
Stabbing pain	20 (19.23)
Stuffy pain	2 (1.92)
Current flowing	4 (3.85)
Burning pain	4 (3.85)
Pain location	
Orbital region	100 (96.15)
Forehead	90 (86.54)
Temporal	88 (84.62)
Vertex	32 (30.77)
Occipital	4 (3.84)
Cheek	2 (1.92)
Teeth	2 (1.92)

Multiple choices were allowed for nature of pain and pain location. VAS, Visual Analog Scale.

**Table 3:** Laterality of headache attacks by sex.

Laterality of headache attacks	Males (*n* = 84)	Females (*n* = 20)	*p* value
Laterality			<0.001
Left-side only	30 (35.71)	14 (70.00)	
Right-side only	54 (64.29)	2 (10.00)	
Alternating bilateral	0 (0)	4 (20.00)	

Data are *n* (%). *p* values were calculated using Fisher’s exact test when the expected frequency in any cell was <5; otherwise, the Chi-square test was used.

### 3.4 Circadian rhythm

Diurnal rhythmicity was observed in a total of 86 patients (82.69%), of whom 51.2% experienced headache attacks across multiple time periods (e.g., 16:00–20:00; 9:00–10:00 plus 13:00–16:00). The predominant time of onset was the afternoon (12:00–17:00), accounting for 51.2%, followed by the morning and evening, each accounting for 34.9% (Figure 3).

**Figure 3:**
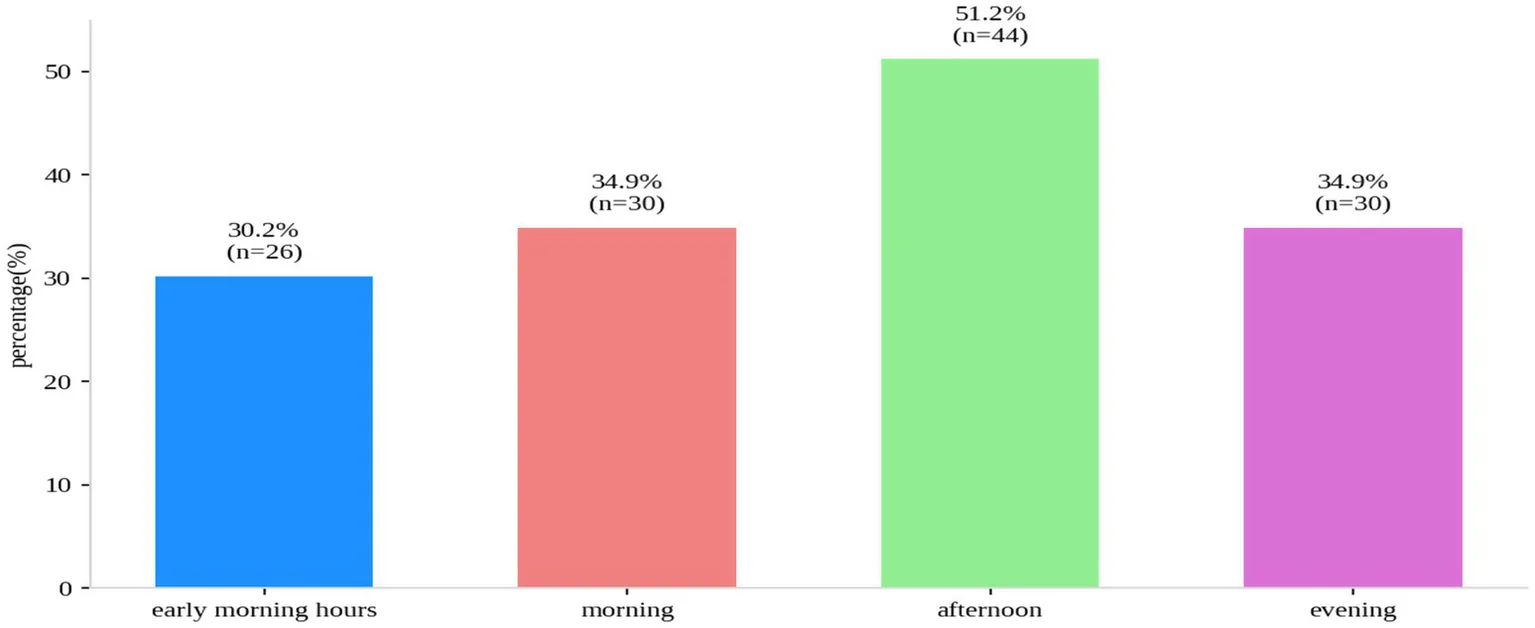
Time of onset attacks in patients with CH. Patients could report multiple time periods; percentages do not sum to 100%.

### 3.5 Frequency and duration of cluster episodes

Among the 100 patients with available data (excluding four patients who presented with their first episode), 50 (50.0%) experienced one cluster episodes per year. The mean duration of cluster episodes was 35.34 ± 25.28 days, with the majority (64.0%) lasting 15–30 d. Strictly unilateral attacks occurred in 96 patients (96.0%), whereas alternating bilateral attacks occurred in four patients (4.0%) across different cluster periods (Table 4).

**Table 4:** Temporal characteristics of cluster periods (*n* = 100).

Temporal characteristics of cluster periods	Statistic
Frequency of cluster periods (times/year)	
≥2	6 (6.00)
1	50 (50.00)
Once / > 2 y	22 (22.00)
Once / 1–2 y	22 (22.00)
Mean duration of cluster periods (days)	35.34 ± 25.28
Laterality alternative in different cluster periods	
Strictly unilateral	96 (96.00)
Alternating bilateral	4 (4.00)
Duration of cluster periods (days)	
≤15	10 (10.00)
16 ~ 30	64 (64.00)
31 ~ 60	22 (22.00)
>60	4 (4.00)

Data are *n* (%) or mean ± SD. *n* = 100 after excluding four patients diagnosed at their first episode.

### 3.6 Cranial autonomic symptoms (CAS)

CAS was observed in 96 patients (92.31%), all of whom had symptoms ipsilateral to the headache. Among the 96 patients with CAS, lacrimation was the most common symptom (68/96, 70.83%), followed by conjunctival injection (58/96, 60.42%). In contrast, eight patients (7.69%) presented without cranial autonomic symptoms during attacks. Among these eight patients, four (50.00%) exhibited agitation accompanied by nausea, two (25.00%) reported agitation with phonophobia and/or photophobia, and the remaining two (25.00%) experienced agitation as the sole accompanying symptom (Table 5).

**Table 5:** CAS and non - CAS in patients with CH.

Accompanying symptoms	Cases (*n*, %)
Laterality of CAS	
Ipsilateral	96 (92.31)
Contralateral	0 (0)
Bilateral	0 (0)
CAS (*n* = 96)	
Conjunctival injection	58 (60.42)
Lacrimation	68 (70.83)
Rhinorrhea	24 (25.00)
Nasal congestion	20 (20.83)
Eyelid edema	12 (12.50)
Ptosis	16 (16.67)
Ear fullness and pain	6 (6.25)
Agitation/restlessness	102 (98.08)
Non-CAS (*n* = 104)	
Nausea	48 (46.15)
Vomiting	6 (5.77)
Phonophobia	8 (7.69)
Photophobia	10 (9.62)
Headache worsens after activity	4 (3.85)
Patients without CAS (*n* = 8)	
Agitation + nausea	4 (50.00)
Agitation + phonophobia/photophobia	2 (25.00)
Agitation only	2 (25.00)

CAS, cranial autonomic symptoms. Percentages for CAS symptoms are based on 96 patients with CAS; percentages for non-CAS symptoms are based on all 104 patients. Multiple choices were allowed.

## 4 Discussion

Our findings closely align with the previously reported clinical features of CH, including male predominance, external triggers, episodic subtype predominance, and characteristic profiles of disease course, diagnostic delay, headache phenotype, and cranial autonomic symptoms (2, 10–13). However, our analysis also identified several notable differences, which are discussed below in the context of specific clinical variables. Notably, although no variable reached statistical significance in the multivariable logistic regression, several exhibited clinically meaningful trends—particularly GAD-7 positivity (OR = 4.32, 95% CI: 0.99–18.86; *p* = 0.051), suggesting a possible association between anxiety and CH, possibly due to the limited sample size.

### 4.1 Sex distribution and demographic characteristics

Among the 104 enrolled patients, eCH accounted for 97.11% and cCH for 2.89%. This episodic predominance is consistent with that of Dong et al. (14), who reported a lower cCH and a higher eCH prevalence in Chinese than in European populations; this may be attributable to racial, lifestyle, or cultural factors (10). Similarly, our study found that Asian patients with CH had lower smoking and drinking rates than Western cohorts, along with a lower daily attack frequency.

Although CH has consistently been reported to be more prevalent in males, recent data indicate a declining male-to-female ratio (15). In South Korea, the ratio decreased from 7.0:1 (10) to 5.4:1 (16). In our study, the male-to-female ratio was 4.2:1, comparable to the 4.33:1 reported by Zhang et al. (17) in a 2024 Chinese study.

A large-scale study in 2012 showed that females aged ≤20 years are more susceptible to CH, whereas males aged 21–30 years are more likely to develop the condition (18). In our study, female patients tended to have an earlier age of onset than males (27.60 ± 11.61 vs. 31.60 ± 8.47 years), although the difference did not reach statistical significance (*p* = 0.326), which may be attributable to the relatively small sample size of female patients.

### 4.2 Smoking and alcohol consumption

In the present cohort, the overall smoking prevalence was relatively low (17.31%, 18/104). This may be explained by several factors. First, our cohort was predominantly composed of patients with eCH (97.11%), and cCH has been associated with a higher smoking prevalence than eCH (11). Second, the relatively high proportion of female patients (20/104) is relevant, as smoking rates among Asian women are generally very low. Third, some patients may reduce smoking to avoid triggering attacks. Previous studies have reported decreased melatonin levels and rhythm disturbances in CH (19), and cadmium and other tobacco compounds may be toxic to the hypothalamic–pituitary-gonadal axis, leading to impaired melatonin secretion and regulation (20). Thus, tobacco may act as a triggering factor for CH attacks, although this remains controversial (20–22). Larger studies are needed to confirm these findings.

Alcohol is recognized as a potential CH trigger by the International Headache Society (IHS) (2). Imai et al. (23) found that 98 of 131 Japanese CH patients consumed alcohol. A Swedish study of 874 CH patients reported that although overall alcohol intake was low, alcohol frequently triggered attacks, particularly in males, contributing to reduced consumption in this population (22). Similarly, only 26.92% patients in our study reported a history of light alcohol consumption, all of whom were male. This finding may be attributable to the fact that alcohol is a well-known trigger for headache, leading to reduced alcohol intake among patients.

### 4.3 Comorbidity

A 2025 U. S. study of 152,727 CH patients reported comorbid depression and anxiety in 28.6 and 14.7%, respectively (24). Fox et al. (25) identified fear of attacks, depression, and anxiety as independent predictors of pain-related disability in 640 CH patients. Jenningsdotter Olofsgård et al. (26) found that 46.5% of CH patients experienced moderate-to-severe insomnia during acute episodes and 43.7% had poor sleep quality, with 22.0% still suffering from insomnia during remission.

In our cohort, the prevalence of depression, anxiety, and sleep disorders was 25%, 32.69%, and 48.08%, respectively, which are consistent with current reports. The underlying mechanisms may involve nocturnal attacks and sleep disorders, recurrent headache-induced fear and stress, hypothalamic dysfunction (8, 27–29), and some researchers have proposed an association with insular lobe function, which is correlated with the severity of depression, and increased insular glucose metabolism may predict depressive symptoms in patients with paroxysmal pain (30). Females demonstrated higher prevalences of depression (50%), anxiety (60%), and sleep disorders (70%) than males, which is consistent with the view that female CH patients are more susceptible to psychiatric comorbidities (11, 18, 31, 32).

### 4.4 Family history

CH has shown significant familial clustering in multiple population-based studies (33), with a reported prevalence range of 0–22% (median 8.2%) (13). A 2022 UK case–control study reported a prevalence of 7.44% among those with a positive family history (34), and a higher proportion of females has been suggested in familial cases (35).

In our cohort, 7.69% of patients had a positive family history, with a higher rate in females (10%) than in males (7.14%), which is consistent with previous findings. All eight affected patients had first-degree relatives with CH. Prior genetic studies have shown that first-degree relatives have a 5- to 18-fold higher risk of developing CH than the general population (36).

Although genetic susceptibility to familial CH has been confirmed (37, 38), the underlying pathogenesis remains unclear and warrants further investigation. Notably, the small sample size of our study limits its generalizability, and larger cohorts are needed to validate these findings.

### 4.5 Misdiagnosis time

A 2012 U. S. study reported that 22% of patients had a misdiagnosis exceeding 10 years, and only 32% received a correct diagnosis within 1 year (18). A 2025 meta-analysis reported a mean diagnostic delay of 10.43 years (95% CI: 9.09–11.77), with a declining trend since the 1960s, particularly after 2000 (39).

In our study, the median time to correct diagnosis (7.0 years) was shorter than the mean diagnostic delay of 10.43 years reported in the 2025 meta-analysis. Nevertheless, 58.9% of patients still received a correct diagnosis more than five years after symptom onset, indicating that the overall delay remains unacceptably prolonged.

### 4.6 Characteristics of individual headache attacks

Although the maximum attack duration in our cohort reached 4 h, exceeding the ICHD-3 typical limit of 180 min, all patients fulfilled all other ICHD-3 criteria for CH and were considered to have an atypical prolonged variant. As noted in the ICHD-3, longer durations may occur as an atypical variant in fewer than half of active episodes. This is consistent with previous observations that prolonged attacks (>180 min) are significantly associated with diagnostic delay (40).

Previous studies have reported that Asian CH patients typically have one to two daily attacks per day, each lasting one to two hours (10). Similarly, once-daily attacks predominated in our study (46.15%), and most patients (84.62%) achieved headache resolution within two hours. Diurnal rhythmicity was observed in 82.69% of patients, consistent with European and American studies (41, 42). The afternoon (12:00–17:00) was the most common onset period (51.2%), corroborating prior findings from China and South Korea (14, 43).

Previous studies (14, 32) have shown that CH is strictly unilateral during cluster periods, with right-sided attacks being more common in both males and females, and that side shifts can occur between or within cluster periods. Similarly, our study found a strict unilateral predominance with a right-sided preponderance. However, female patients tended to have left-sided attacks, and bilateral alternation across cluster periods was observed. In contrast to previous reports, left-sided headaches predominated among female patients in our cohort. This discrepancy may be attributable to the small number of female patients (*n* = 20), and larger studies are needed to verify these findings.

In our study, the distribution of the most common pain locations was consistent with that reported in a 2024 U. S. study (44). In addition, pain at sites not included in the ICHD-3 criteria, such as the cheeks and teeth, was also observed.

Of all patients, 8 exhibited exclusive non-autonomic symptoms (agitation, nausea, and phonophobia/photophobia) in the absence of autonomic involvement, and agitation was the sole symptom shared across this subset. Previous studies have reported that a minority of cluster headache patients present without cranial autonomic symptoms during attacks. For instance, a 2021 Korean multicenter study identified that 19 of 216 patients (8.8%) lacked these autonomic features, suggesting that the absence of cranial autonomic symptoms does not exclude the diagnosis of cluster headache (48). Given that CAS may overlap with migraine and even occur in frequent episodic tension-type headache (45), clinicians should remain vigilant to avoid misdiagnosis, particularly in atypical presentations without autonomic features.

### 4.7 Time regularity of the cluster period

In our study, 50.00% of patients experienced one cluster episode per year. The mean cluster period duration was 35.34 ± 25.28 days, with the majority (64%) lasting 15 ~ 30 days. These findings are consistent with a 2024 Chinese study of 816 CH patients (17) and a 2023 South Korean review of CH studies across different countries (15).

In this study, the circadian peak occurred in the afternoon. This may be explained by the hypothesis that the left and right suprachiasmatic nuclei (SCN) constitute antiphase-coupled oscillators, whereby abnormal activation of this “split state” in CH may contribute to both headache laterality and a “phase shift” in rhythm—displacing attacks from the nocturnal window to the daytime (46). Alternatively, the “residual effect” of OSA proposed by Chervin et al. (2000) may contribute, although OSA was not documented here and will be addressed in future questionnaires (47). Moreover, a 2023 Korean multicenter study showed that circadian rhythmicity evolves with disease progression, gradually dichotomizing into “hypnic” and “midday” patterns (15). Thus, the afternoon predominance in our cohort may reflect an evolving rhythm pattern rather than a fixed subtype.

Several limitations should be acknowledged. First, the cross-sectional design relied on patient self-reports for data on age at onset, attack frequency, lifestyle habits, and misdiagnosis, which may introduce recall bias. However, the severe nature of CH likely facilitates accurate recall of attack characteristics, partially mitigating this bias. Second, the single-center design and modest sample size limit the generalizability of our findings. The small female subgroup (*n* = 20) may have further reduced statistical power to detect sex differences, particularly in age at onset. Future multicenter studies with larger, more balanced cohorts are warranted to validate our observations. Accordingly, enhanced clinical surveillance with regular follow-up is recommended to observe dynamic changes in the features of cluster periods over time.

## 5 Conclusion

In summary, this study is the first to comprehensively characterize the clinical phenotype of CH in the Dalian region. Our findings are largely consistent with previous domestic and international studies, revealing several sex-specific features: female patients tend to have an earlier age of onset, several present with atypical cranial autonomic symptoms (CAS), and side shifts of headache attacks occur exclusively in females—observations that align with recent Chinese studies. However, due to the lack of genetic testing, we were unable to assess the hereditary contributions to CH in this cohort.

## Data Availability

The original contributions presented in the study are included in the article/supplementary material, further inquiries can be directed to the corresponding author.
